# Fusion of Protein Aggregates Facilitates Asymmetric Damage Segregation

**DOI:** 10.1371/journal.pbio.1001886

**Published:** 2014-06-17

**Authors:** Miguel Coelho, Steven J. Lade, Simon Alberti, Thilo Gross, Iva M. Tolić

**Affiliations:** 1 Max Planck Institute of Molecular Cell Biology and Genetics, Dresden, Germany; 2 FAS Center for Systems Biology, Harvard University, Cambridge, Massachusetts, United States of America; 3 Max Planck Institute for the Physics of Complex Systems, Dresden, Germany; 4 Stockholm Resilience Centre, Stockholm University, Stockholm, Sweden; 5 Department of Engineering Mathematics, Merchant Venturers School of Engineering, University of Bristol, Bristol, United Kingdom; 6 Division of Molecular Biology, Ruđer Bošković Institute, Zagreb, Croatia; University of California San Francisco, United States of America

## Abstract

Asymmetric segregation of damaged proteins at cell division generates a cell that retains damage and a clean cell that supports population survival. In cells that divide asymmetrically, such as *Saccharomyces cerevisiae*, segregation of damaged proteins is achieved by retention and active transport. We have previously shown that in the symmetrically dividing *Schizosaccharomyces pombe* there is a transition between symmetric and asymmetric segregation of damaged proteins. Yet how this transition and generation of damage-free cells are achieved remained unknown. Here, by combining *in vivo* imaging of Hsp104-associated aggregates, a form of damage, with mathematical modeling, we find that fusion of protein aggregates facilitates asymmetric segregation. Our model predicts that, after stress, the increased number of aggregates fuse into a single large unit, which is inherited asymmetrically by one daughter cell, whereas the other one is born clean. We experimentally confirmed that fusion increases segregation asymmetry, for a range of stresses, and identified Hsp16 as a fusion factor. Our work shows that fusion of protein aggregates promotes the formation of damage-free cells. Fusion of cellular factors may represent a general mechanism for their asymmetric segregation at division.

## Abstract 2

Fusion of harmful aggregated proteins into larger clumps increases the asymmetry of segregation of damage at cell division, favoring the production of rejuvenated cells.

## Author Summary

During their lifetime, cells accumulate damage that is inherited by the daughter cells when the mother cell divides. The amount of inherited damage determines how long the daughter cell will live and how fast it will age. We have discovered fusion of protein aggregates as a new strategy that cells use to apportion damage asymmetrically during division. By combining live-cell imaging with a mathematical model, we show that fission yeast cells divide the damage equally between the two daughter cells, but only as long as the amount of damage is low and harmless. However, when the cells are stressed and the damage accumulates to higher levels, the aggregated proteins fuse into a single clump, which is then inherited by one daughter cell, while the other cell is born clean. This form of damage control may be a universal survival strategy for a range of cell types, including stem cells, germ cells, and cancer cells.

## Introduction

A dividing cell can deal with damaged material in two different ways. First, the damaged material can be segregated asymmetrically during division, such that it is concentrated in one of the two newborn daughter cells, while the other cell is born clean. The damage is then removed from the population when the cell retaining the damaged material dies. Second, in phases of rapid growth, damaged material can be segregated randomly, leading to less asymmetric segregation of damage between daughters. In this case, accumulation of damage within any cell is prevented by rapid divisions that dilute the damaged material.

Protein aggregates are a type of damaged material, composed of insoluble and dense protein particles [1]. These aggregates, instead of being degraded, accumulate in the cell during stress and aging [2]–[4]. Once formed, aggregates can interfere with cell cycle progression and cell function [5] and correlate with cell death [6]. To deal with protein aggregates during cell division, *Escherichia coli* and *Saccharomyces cerevisiae*, as well as stem cells, use asymmetric segregation, where aggregates are retained by one cell, generating a clean sister cell [2],[3],[7]–[10]. In *E. coli*, protein aggregates accumulate at the cell poles and often segregate with the old cell pole [3]. In the case of *S. cerevisiae*, asymmetric segregation of aggregates is achieved through a combination of retention in specialized compartments [8],[11]–[14], active transport [8], and limited diffusion through the bud neck [9]. However, the mechanisms underlying aggregate segregation in eukaryotic cells that divide symmetrically are unclear.

We have recently shown that the symmetrically dividing fission yeast *Schizosaccharomyces pombe* does not show aging under favorable conditions, which suggests that aggregates are segregated symmetrically [6]. After stress, however, the cells inheriting large aggregates do age and eventually die, while their sisters with small or no aggregates do not age [6]. How a large aggregate arises after stress, and how the generation of aggregate-free cells is achieved, remained unknown.

Here we study the mechanism underlying the transition from symmetric to asymmetric aggregate segregation. By combining *in vivo* quantification of aggregate nucleation, movement, fusion, and segregation with a mathematical model, we show that under favorable conditions aggregates fuse rarely and segregate symmetrically at division. Using the total amount of aggregates, measured as the total fluorescence intensity in puncta of the GFP-tagged Hsp104 disaggregase [6], to identify different levels of aggregation in response to stress, our experiments show that an increase in fusion facilitates asymmetric segregation of aggregates and production of aggregate-free cells. These results are consistent with the predictions of our model, which provides support for the conclusion that the formation of damage-free cells is promoted by aggregate fusion.

## Results

### Protein Aggregate Dynamics *in Vivo*: Nucleation, Movement, and Fusion

**Figure 1:**
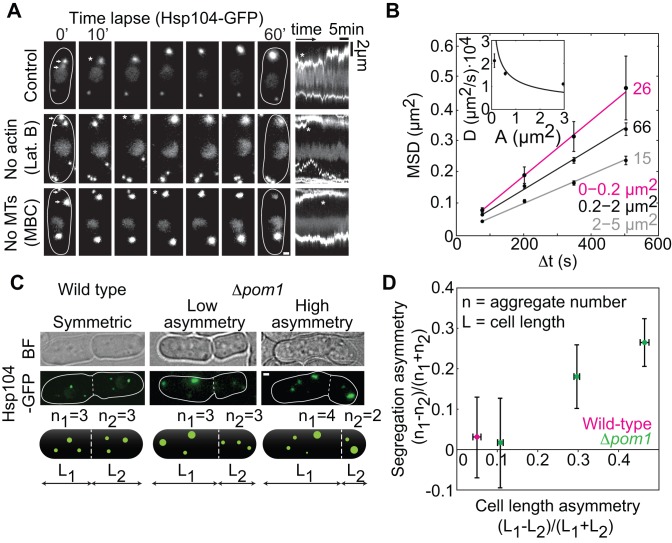
Aggregates move by diffusion and segregate proportionally to the cell volume. (A) Aggregate movement and fusion in control cells and after cytoskeleton depolymerization (Latrunculin B and MBC); kymographs (space-time plots, right). The fusion of two aggregates (arrows at 0′) is marked by an asterisk. (B) Mean squared displacement (MSD) of aggregates in control cells grouped by area (see labels) and a weighted fit *MSD = *4*D*Δ*t* + offset (lines). (Inset) Diffusion coefficient, *D*, versus area, *A*, weighted fit proportional to 1*/√A* as expected from Stokes diffusion. Fitting with a nonlinear equation (*MSD = v^2^*(Δ*t*)*^2^ +* 4*D*Δ*t +* offset) yielded a worse fit (parameter-adjusted r^2^, linear_(2–5 µm2)_ = 0.996, nonlinear_(2–5 µm2)_ = 0.969). (C) Aggregate segregation (|n_1_−n_2_|) at division in wild type and Δ*pom1*. (D) Aggregate segregation asymmetry versus cell length asymmetry (*n*>30 cells/data point). The data are mean ± SEM from >3 independent experiments; scale bars, 1 µm. See also Figures S1 and S2.

We monitored protein aggregates using the Hsp104 disaggregase, a chaperone that binds and separates aggregated proteins [15], labeled with GFP (Figure 1A, Figure S1, and Text S1). We have shown before that Hsp104 from *S. pombe* is active as a disaggregase *in vitro* and *in vivo* [6] and that the puncta of Hsp104-GFP observed in the cytoplasm represent endogenous aggregates. We also observed diffuse Hsp104-GFP in the nucleus (Figure 1A) and in the cytoplasm (see Figure S2F), as shown previously in *S. cerevisiae* [16]. While the lower disaggregase activity of Hsp104 from *S. pombe*, when compared to its *S. cerevisiae* homolog [6], likely accounts for the presence of aggregates under favorable conditions, deleting hsp104 resulted in increased aggregation (Figure S1F–I) and increased cell death after stress [6], while labeling the endogenous Hsp104 with GFP has no effect on stress recovery [6]. The Hsp104-GFP puncta are composed of aggregated proteins and chaperones (Figure S1), as reported for other organisms [5].

To study aggregate dynamics during the cell cycle, we followed Hsp104-associated aggregates with wide-field fluorescence microscopy (Materials and Methods). Aggregates nucleated equally often in each of the two respective cytoplasmic regions (compartments) between the nucleus and the old cell pole, and the nucleus and the new cell pole, generated in the previous division (1.3±0.2 nucleation events/cell cycle, *n* = 162 cells; Figure S2A and S2B). After nucleation, aggregates typically remained in the same compartment (only 3.2±1.5% of aggregates moved between the compartments, *n* = 126 cells). Aggregates moved and contact between them resulted in their fusion (94/100 contacts resulted in fusion; 0.40±0.06 fusion events/cell cycle, *n* = 200 cells; Figure 1A, Movie S1). Fission of aggregates was rare (0.006±0.005 events/cell cycle), and disappearance of aggregates was not observed (*n* = 498 cells). We tracked individual aggregates on time scales from milliseconds to tens of minutes and observed dynamics suggesting diffusive motion (Figures 1B and S2C–F and Movie S2). To test whether aggregate movement was diffusive and not linked with the movement of other subcellular components, we performed a combination of tests, which confirmed that aggregates (1) move according to Stokes diffusion (Figure 1B, inset), (2) do not co-localize with the cytoskeleton (actin or microtubules) or a wide range of lipid structures (cellular membrane, endosomes, Golgi, vacuoles, and nuclear membrane) (Figure S2G and S2H), and (3) still undergo diffusion and fusion when the cytoskeleton is depolymerized (Figures S2I–K; see also Text S1).

We next studied how aggregates are segregated between cells at division. Because aggregates nucleate and move randomly, we hypothesized that sister cells arising from a morphologically symmetrical division inherit the same number of aggregates on average. Indeed, the aggregates did not segregate specifically to a cell inheriting the new or the old pole (Figure S2B; the small bias can be a consequence of the displacement of aggregates towards the old pole by the nucleus during anaphase). In the wild type, the two equally sized sister cells inherited on average the same number of aggregates (Figure 1C and 1D). Because asymmetric cell division may lead to biased segregation of aggregates towards the larger sister cell, we enforced asymmetry in cell division by using a Δ*pom1* mutant, in which the division plane is displaced off-center, resulting in two cells of different size [17]. We observed that cells were up to 70% larger than their smaller sisters, and larger cells retained correspondingly more aggregates (Figure 1C and 1D and Movie S3). These results show that aggregate segregation in *S. pombe* is unbiased. We conclude that aggregate nucleation and movement is random, resulting in random aggregate segregation at division.

### The Stochastic Aggregation Model Predicts Unbiased Aggregate Segregation at Cell Division

**Figure 2:**
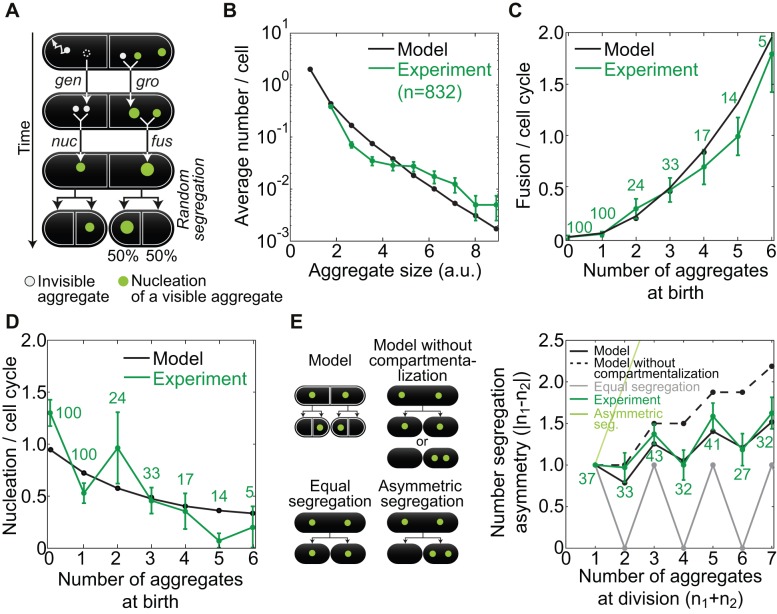
A stochastic model for aggregate dynamics and segregation at cell division. (A) The model. Smallest size aggregates (gray) are generated (*gen*) and fuse, resulting in nucleation (*nuc*) of an aggregate of size ≥ *ν* (green, *ν = *visibility threshold), fusion events (*fus*) between aggregates of size ≥ *ν*, and growth (*gro*) of an aggregate of size ≥ *ν* due to fusion with an aggregate of size < *ν*. Aggregates are randomly assigned to one of the two new compartments at division. (B) Aggregate size distribution (a.u.) averaged across the cell population. Aggregate amount from the experiment was compared with aggregate sizes from the model by scaling, with a scaling parameter *I*_0_ (Figure S3A). (C) Number of fusion events per cell cycle versus number of aggregates at birth, in model and experiment (see labels). (D) Nucleation events per cell cycle as a function of the number of aggregates at birth, in model and experiment (see labels). (E) Segregation asymmetry of aggregate number versus the total number of aggregates at division (see text). The scheme on the left represents different modes of segregation. The data are mean ± SEM from >3 independent experiments. See also Figure S3.

Based on our experimental observations, we developed a stochastic aggregation model (Figure 2A) that allows for the simulation of aggregate size distributions (Figure 2B), which can be compared with the experimentally observed size distributions (measured by the intensity of Hsp104-GFP in each puncta, a.u.). A key feature distinguishing the proposed model from other models [18]–[20] is that aggregate segregation asymmetry is an output rather than an input of our model.

Three key processes operate on size distributions of aggregates in each of the two compartments of a cell (Figure 2A): (1) generation of the smallest size aggregates at rate *r*; (2) fusion of aggregates of sizes *i* and *j* at rate *K*(*i*,*j*) to create an aggregate of size *i*+*j*; and (3) random segregation of aggregates to two new compartments at division. We use the Brownian kernel:
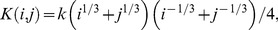
(1)where *k* = *K*(1,1) is a parameter to be determined. This well-established kernel [21],[22] can be derived from Brownian diffusion of aggregates with Stokes friction, a fusion rate increasing in proportion to the sum of the aggregates' radii, and aggregate packing such that size (volume) is proportional to radius cubed. In this manner, the effect of spatial diffusion on fusion rate is incorporated into the model, without explicitly simulating spatial diffusion [9]. We introduce a visibility threshold *ν* below which aggregates cannot be detected by wide-field fluorescence imaging (Figure S3A). A visible nucleation event occurs when two nondetectable aggregates fuse, forming a detectable one.

Generation and fusion of aggregates within compartments were simulated with a stochastic aggregation algorithm [23], which in turn was embedded within another algorithm that implemented random aggregate segregation among compartments at division (Text S1). The testable predictions of our model are (i) large aggregates are rare, while small ones are more abundant; (ii) an increase in the number of aggregates at cell birth gives rise to a decrease in aggregate nucleation and (iii) to an increase in fusion; (iv) at cell division, the pattern of aggregate segregation into the daughter cells is between a completely symmetric and a random one; and (v) aggregate fusion increases their segregation asymmetry at cell division and promotes the birth of aggregate-free cells. These model predictions are general features of the model behavior and are not dependent on specific parameter values. We will now compare predictions i–iv with our experimental results. Prediction v will be tested in the response-to-stress extension of the model described below.

The experimentally measured size distribution of aggregates shows that small aggregates are found more frequently than large ones (Figure 2B), confirming prediction i. Whereas the experimentally measured number of fusion events increases with the total number of aggregates (Figure 2C), the number of nucleation events shows the opposite trend (Figure 2D), confirming predictions ii and iii. The model therefore shows that in the presence of a high number of visible aggregates, an invisible aggregate is increasingly likely to fuse with a visible aggregate rather than fusing with another invisible aggregate to create a visible aggregate, which is observed as nucleation. Parameter values were then fitted (Figure S3A) to obtain quantitative as well as qualitative consistency for these three predictions (Text S1). The parameter values were additionally corroborated by theoretical arguments (Text S1).

The parameterized model predicts a pattern of aggregate segregation at cell division by aggregate number that is between completely symmetric segregation, where the difference in the aggregate number is the minimal possible, and fully random segregation, where each aggregate can segregate to either of the two newborn cells, corresponding to the model without compartmentalization. The experimentally measured segregation pattern closely matches that predicted by the model, thereby confirming prediction iv (Figure 2E). Thus, our results do not support a biased segregation (by compartment) of aggregates in fission yeast.

### Aggregates Fuse Before Segregating Asymmetrically Under Stress Conditions

If the average aggregate amount formed per cell cycle is substantially less than the amount which affects cell growth (death threshold “*d*”, 5 a.u.) [6], symmetric segregation at division is sufficient to dilute the aggregates and allow survival, but if the average amount is more than what would be required to kill both daughter cells, asymmetric segregation may be necessary for one of the daughter cells to survive.

**Figure 3:**
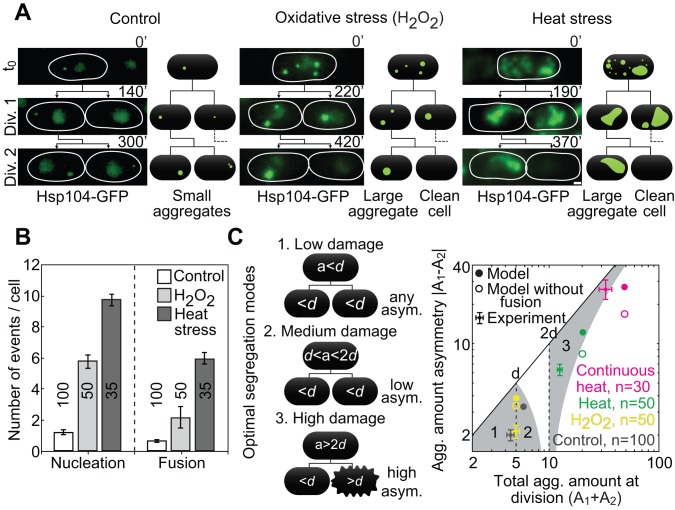
A transition to asymmetric segregation after stress. (A) Aggregate segregation after stress (Hsp104-GFP, see labels). Thin lines encircle cells; scale bar, 1 µm. (B) Aggregate nucleation and fusion during the first cell cycle after stress. (C, Left) Three distinct optimal segregation regimes (1–3) that maximize the number of surviving cells depending on the aggregate amount in the mother cell at division (a): (1) any segregation asymmetry when the total aggregate amount is below the death threshold (*d*), (2) low segregation asymmetry when the amount is between *d* and 2*d*, and (3) high asymmetry when the amount is above 2*d*. Black lines represent pedigree lineage, and the rough cell edge represents cell death (cell lysis and shrinkage, followed by absence of growth). (Right) Aggregate amount asymmetry (absolute difference in the amount between sister cells at birth) under favorable conditions (“Control”) and at division 2 after stress: 1 mM H_2_O_2_ for 1 h (“H_2_O_2_”), 40°C for 30 min (“Heat”), continuous growth at 40°C (“Continuous heat”), and the model (see labels). Grey areas numbered 1–3 are the optimal segregation regions (see scheme and text). The data are mean ± SEM; number of cells from >3 independent experiments are given in graphs. See also Figure S4.

We tested the effect of a range of aggregate levels on segregation dynamics and on cell viability. To increase the aggregate amount, we used stress conditions such as oxidative stress (H_2_O_2_) and transient or continuous heat stress (T = 40°C) (Figure 3A). Both types of stress increased the number of aggregate nucleation and fusion events (Figure 3B). As in the control situation, aggregate movement after heat stress was consistent with Stokes diffusion (Figure S4A and S4B) and 97 out of 103 aggregate contacts resulted in fusion. During recovery from stress, aggregates did not co-localize significantly with actin structures or microtubules (Figure S4C). As under control conditions (Figure S2J), nucleation and fusion of aggregates after stress occurred also in the absence of actin or microtubule structures (for cells treated with Lat.B or MBC, 94/102 or 90/97 contacts resulted in fusion, respectively; Figure S4D). Remarkably, fusion converted the aggregates into a single large one within the first few cell cycles after stress (Figure 3A). This single aggregate was asymmetrically segregated to one of the sister cells at division (Figure 3A), while the other sister cell was born without aggregates (segregation was not biased towards the old or the new cell pole; Figure S4E). Cells with an aggregate amount greater than *d* typically died (28/49 cells), whereas their sisters survived (48/49 cells), indicating that the clearance of aggregates through asymmetric segregation is important for viability. To address whether the aggregate number has an effect on the cell cycle [7] of cells born with similar aggregate amounts, we compared the division time of cells inheriting only one aggregate with that of cells inheriting two or more aggregates (Figure S4F). We observed no significant difference in the division time of cells containing one or more aggregates (Figure S4F), which agrees with our previous observation that the total aggregate amount correlates more strongly with cell death than aggregate number [6].

To test whether the transition to asymmetric segregation could be reproduced theoretically, we introduced stress into the model, using the parameters fitted for control conditions. We raised the aggregate generation rate *r* to obtain the experimentally observed aggregate nucleation upon heat stress (Figure 3B) in one simulated cell cycle, and then returned *r* to the control value and simulated for another cycle before the first cell division (*r* values are shown in Figure S3A), to account for the duration of the experimental stress recovery. The experimentally observed size distributions (Figure S4G), dependence of fusion on the number of aggregates (Figure S4H), and aggregate segregation patterns (Figure S4I) were consistent with the model including stress, indicating that the model is robust. The model shows a 10-fold increase in the number of fusion events compared to the control situation, which is explained by the increased aggregate number (Figure S4H). Fusion causes a shift toward large aggregate sizes after stress, and faster recovery to the control size distribution for small aggregate sizes at division 2, in both model predictions and experimental results (Figure S4G). Thus, the stochastic aggregation model is consistent with the observed aggregate behavior after stress.

To understand which segregation modes maximize daughter cell survival for a given total aggregate amount, we model the effect of the segregation asymmetry on cell survival by assuming that, as observed experimentally [6], a cell dies if it has a total aggregate amount at birth above the death threshold *d*. This leads to three distinct optimal segregation regimes that maximize the number of surviving cells: (1) any segregation asymmetry when the total aggregate amount at division is below *d*, (2) low segregation asymmetry when the amount is between *d* and 2*d*, and (3) high asymmetry when the amount is above 2*d* (Figure 3C, scheme and corresponding gray regions in graph).

**Figure 4:**
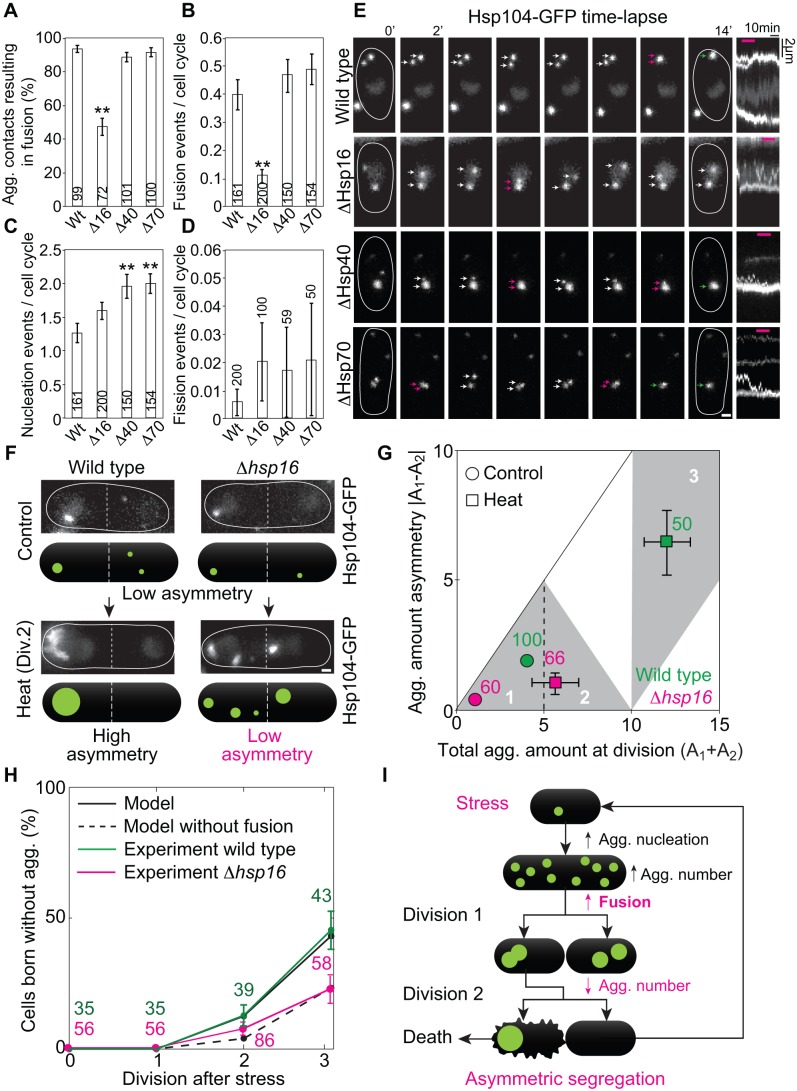
Hsp16 is required for efficient aggregate fusion, which facilitates asymmetric segregation and generation of cells clean of aggregates. (A) Quantification of the percentage of contact events between two aggregates which resulted in fusion. Nucleation (B), fusion (C), and fission (D) events per cell cycle in the wild-type strain and in the strains in which Hsp16, Hsp40, or Hsp70 was deleted (statistical difference between wild type and mutants: **p<0.01). (E) Time-lapse of aggregate movement in the wild type and the strain where Hsp16, Hsp40, or Hsp70 was deleted (arrows mark aggregates, magenta indicates contact between aggregates, green corresponds to fusion). In the kymographs, fusion events are visible as the merging of two aggregates (two traces merge into one thicker trace; magenta lines on top correspond to time interval depicted in panels). In the *Δ*hsp16 mutant, a contact event does not give rise to fusion, and the aggregates remain separated. (F) Aggregate segregation in wild-type and hsp16 deleted strains, under control and heat stress conditions. (G) Aggregate amount of asymmetry in wild-type and a Δhsp16 strain (in which fusion was reduced; see labels and regions 1–3 depicted in Figure 3C). Error bars on the control data are not visible because they are smaller than the circles representing the data. (H) Fraction of cells born without aggregates at the first three divisions after stress (see labels). The data in (A–D), (G), and (H) are mean ± SEM; number of aggregate contact events or cell cycles from >3 independent experiments are given in the graphs. In (E) and (F) thin lines encircle cells; scale bars, 1 µm. (I) Summary: stress increases nucleation of aggregates, leading to an increased number of aggregates per cell. Fusion decreases the total aggregate number to a single large aggregate, forcing its asymmetric segregation. This results in the birth of a clean cell. See also Figure S4.

The model predicts that fusion facilitates asymmetric segregation in response to different levels of stress, where high asymmetry is optimal (Figure 4C, filled circles). This behavior was also observed experimentally for a range of stresses (Figure 4C, filled squares). We observed that in divisions 2 and 3 after stress, the percentage of cells born without aggregates was higher for stress conditions that originated in a higher aggregate amount (Figure S4J). This phenomenon can be explained by the higher number of fusion events observed for high stress levels (e.g., heat stress as opposed to oxidative stress; Figure 3B), which can result in the faster generation of a single large aggregate. Once large aggregates are formed, nucleation of aggregates decreases in favor of the growth of the large aggregates: as observed for unstressed cells (Figure S4C), the nonvisible aggregates have a higher probability to fuse with large preexisting aggregates. We conclude that in response to increased aggregate amount, an increase in fusion leads to fewer aggregates and thus more asymmetric segregation, which promotes the formation of aggregate-free cells.

### Hsp16-Mediated Aggregate Fusion Facilitates Asymmetric Segregation

The model predicts that reducing fusion decreases segregation asymmetry (Figure 3C, empty circles). To test the prediction, we needed to identify a molecular factor that would reduce fusion. Small heat shock proteins are a special class of chaperones, which bind and sequester misfolded proteins [24]. The fission yeast small heat-shock protein 16 (Hsp16) was described to co-aggregate with misfolded proteins during stress [25]; therefore, we hypothesized that Hsp16 has a role in the fusion of aggregated proteins *in vivo*. Indeed, we observed that when we deleted Hsp16, the number of aggregate contacts resulting in fusion decreased (Figure 4A and 4E) and aggregate fusion per cell cycle also decreased (Figure 4B), which correlated with an increase in the number of cells containing aggregates in the population (Figure S4M). Aggregate nucleation (Figure 4C) and fission (Figure 4D) per cell cycle was not significantly altered in the absence of Hsp16. The total amount of aggregates was unaffected by the deletion of Hsp16 (Figure S4L), which argues against the possibility that in the absence of Hsp16 there are generally more damaged proteins. Thus, Hsp16 is primarily an aggregate fusion factor.

The decrease in fusion efficiency was specific to Hsp16 deletion, as deleting Hsp40 or Hsp70, molecular chaperones that participate in protein disaggregation [26], did not interfere with fusion or fission significantly (Figure 4A, 4B, and 4D). Contrary to Hsp16 deletion, deleting Hsp40 or Hsp70 caused an increase in aggregate nucleation (Figure 4C) and total amount per cell (Figure S4L), whereas an increase in total aggregate number per cell was observed in all three deletions (Figure S4M). Taken together, these results suggest that the increase in the number of aggregates in Δhsp16 cells compared to the wild type is a consequence of reduced fusion.

We proceeded to test the prediction of the model in the strain deleted for Hsp16. We observed that decrease in fusion resulted in a decrease in the segregation asymmetry of aggregate amount (Figure 4F and 4G), as expected from the model where, as a qualitative approximation, aggregates were not allowed to fuse after stress (Figure 3C). The model including aggregate fusion also precisely predicted the fraction of cells born without stress-induced aggregates at each division after stress in the wild type (Figure 4H). Remarkably, in spite of the fact that 10 aggregates on average were formed after stress (Figure 3B), by the second and third division, ∼15% and 50% of the cells were born clean of aggregates, respectively (Figure 4H). Importantly, when the aggregates were not allowed to fuse in the model including stress, the fraction of cells born free of aggregates was halved (Figure 4H). Parameter sensitivity analysis shows that the fraction of cells born clean after stress is highly sensitive to the strength of the fusion process during recovery (k), and is also decreased by a faster generation of aggregates (r) during stress (Figure S3B), as would be intuitively expected. The average number of aggregates per cell immediately after stress is increased by generation during stress (r) and decreased by fusion combining aggregates together (k) (Figure S3C). Both the fraction of cells born clean and the number of aggregates after stress are insensitive to the generation rate and fusion rate before stress was applied, as well as to the number of aggregates with which the first cells in the simulations were initialized.

As predicted by the model without fusion, we observed in the experiments a ∼50% decrease in the fraction of aggregate-free cells in Δhsp16 compared to wild-type cells (Figure 4H), which correlated with an increase in the fraction of dead cells after heat stress (17±2% in Δhsp16 versus 9±1% in wild type, mean ± SEM, *n* = 123 and 140 cells, respectively). We conclude that fusion facilitates asymmetric damage segregation and accelerates the generation of cells clean of stress-induced aggregates, as stated in prediction v described above.

## Discussion

In this study, we show that fusion of aggregated proteins into a single large unit is sufficient to establish asymmetric segregation of damage, thereby generating a cell clean of aggregates. Below we explore how fusion compares to other mechanisms described to establish asymmetric segregation at cell division, and how fusion might represent a general strategy for asymmetric segregation of cellular components.

### Asymmetry in Response to Damage

We have demonstrated that the symmetrically dividing cells of *S. pombe* undergo a transition to highly asymmetric segregation of protein aggregates, which is facilitated by aggregate fusion. As we observed that aggregates occur in the absence of Hsp104, both under favorable and stress conditions (Figure S1F–H), fusion is likely occurring for aggregated proteins in general, and is not specific of Hsp104-associated aggregates.

In response to increased aggregate nucleation, two distinct mechanisms—stochastic movement and chaperone-mediated fusion of aggregates—combine to generate a single large unit of damage, which has to be segregated asymmetrically, resulting in the birth of a damage-free cell (Figure 4I). Creation of a single large unit requires extensive fusion, which is promoted by an increase in the number of aggregates and a higher Hsp16 chaperone level (Figure S1F), as a consequence of heat stress [27]. It is possible that fusion has a cytoprotective effect [28] by merging the aggregates in a single unit, such as during the first two cell cycles following stress recovery, before a clean cell is born. However, irrespective of the number of aggregates, if the cell is born with a total aggregate amount above the death threshold, this cell is likely to die [6].

Due to the geometry of cell division in *S. pombe*, the asymmetry in segregation can only be established at the second division after stress. This becomes clear when considering the extreme scenario where all aggregates fuse into a unit in both cell compartments within the first cell cycle after stress. In this case, each sibling receives one large aggregate after the first division. In the second division, 50% of cells inherit this single aggregate, while their sisters are born clean. This, however, occurred only in a smaller percentage of the cells. The cells took, on average, one extra cell cycle to generate an aggregate-free cell, at the third division. This delay may be because the frequency of aggregate fusion events decreases over the first and second division, as the total number of aggregates is reduced. It is likely that the activated stress response promotes survival of cells with a high total aggregate amount for more than two divisions after stress, to ensure survival until cells with nonlethal amounts of aggregates are generated.

### Fusion as a Conserved Mechanism of Damage Segregation

How do protein aggregate dynamics and segregation in *S. pombe* compare to those in other organisms? In *S. cerevisiae* and in kidney and ovary cells, aggregates are anchored to or transported by the cytoskeleton [8],[10],[29],[30] and localize to functionally distinct protein quality control compartments [11],[13],[31],[32], which may also be involved in the asymmetric segregation of aggregates [11],[12]. In budding yeast, the sorting of misfolded proteins into these compartments is dependent on a small heat-shock protein, Hsp42 [12],[14],[31]. Hsp42 carries an N-terminal extension, which may promote anchoring of aggregates to the cytoskeleton [14] or membrane compartments [11], thus ensuring their selective retention in the mother cell. Small heat-shock proteins in *S. pombe*, however, lack this N-terminal domain and do not interact with the cytoskeleton or organelles, which agrees with our observation that aggregate movement is random. The specific role of Hsp16 in aggregate fusion and cell survival after stress [6] suggests that fusion is a regulated process that is essential for the cell, as opposed to the consequence of an unregulated aggregate seeding process, observed in cells lacking Hsp40 or Hsp70. Taken together, these findings suggest that an organisms' mode of cell division—morphologically symmetric versus asymmetric—generates specific evolutionary constraints, which may be counterbalanced by the invention or refinement of molecular pathways for concentrating and inheriting protein aggregates.

While in *S. cerevisiae* [11]–[13] and mammalian neurons [29] aggregates associate with subcellular structures, in *E. coli* and neuroblast cells aggregates localize to nucleoid-free [33] or organelle-free cytoplasmic regions [34], respectively. A common aspect of aggregate behavior in all these different systems is movement—either by diffusion [9],[28],[31],[35] or active transport [8],[29]—which may allow for contacts and fusion between aggregates to occur. Therefore, fusion might be a conserved mechanism that contributes to asymmetric segregation of aggregates.

### Fusion as a General Mechanism for Asymmetry

Fusing a number of molecules/components in a cell represents an opportunity to segregate asymmetrically. In mathematical terms, fusion increases the difference between the number of aggregates inherited by daughter cells at segregation. While low numbers of a component that is randomly segregated at division assures a higher variability in individual cells in the population, the formation of a unitary component assures a complete asymmetry in segregation that might be important when minimizing damage or maximizing resources. Fusion might also be a mechanism to establish asymmetry in the localization of aggregated functional molecules within the cell [36],[37], as an increase in the size of the aggregate will lower its diffusion or cause it to be physically trapped between large organelles. The concept of fusion as a mechanism to achieve asymmetry may extend to other phase-partitioned molecules, such as prions [38], metabolic enzymes [39],[40], or RNA granules [41]. In general, fusion of cellular factors may represent a general mechanism to achieve asymmetric localization and segregation at cell division.

## Materials and Methods

### Fission Yeast Culture and Genetic Manipulation

Cells were grown as described before [42]. For imaging, cells were transferred to a MatTek dish (MatTek, Ashland, USA) and imaged in liquid media (YE5 or EMM) or covered with a solid agarose pad (YE5-4% Agarose, SeaKem, Hessisch Oldendorf, Germany) at 30°C. For stress resistance, assays cells were treated with water, as a control, or oxidative stress with 1 mM H_2_O_2_ (Sigma-Aldrich, Hannover, Germany) followed by growth at T = 30°C (70% of cells undergo mitosis, *n* = 30), heat stress of 40°C for 30 min followed by growth at T = 30°C (67% of cells undergo mitosis, *n* = 30), or continuous heat (stress of 40°C for 1 h followed by growth at 37°C, 53% of cells undergo mitosis, *n* = 30). Under favorable conditions, 99.7% of cell complete mitosis successfully [6]. Strains were constructed using a PCR-based gene targeting technique [43], where the label was inserted in the C-terminal region of the target gene in the native genomic locus, keeping it under the control of native expression regulators.

### Imaging Protein Aggregates Labeled with Hsp104-GFP

Cells were imaged in a DeltaVision core microscope, with a motorized XYZ stage (AppliedPrecision, USA). An Olympus UPlanSApo 100× 1.4 NA Oil (R.I. 1.516) immersion objective was used (Olympus, Tokyo, Japan). The illumination was provided by a LED (transmitted light) and Lumicore solid-state illuminator (SSI-Lumencore, fluorescence), and the images were acquired with a Cool Snap HQ2 camera (Photometrics, Tucson, AZ, USA) and the SoftWorx software (AppliedPrecision, USA), using 2×2 pixel binning, to minimize light exposure (pixel size = 0.1288 µm). For long-term time lapse imaging, Z-stacks for 6–12 nonoverlapping imaging areas in the sample were acquired every 10 min (total time = 20 h) and in short time-lapses every minute (total time = 1–3 h). For single Z-stacks cells were imaged with exposure = 0.05–0.20 s, 2%–50% transmission, depending on the protein and fluorescent label. As a control for photo-toxicity, cell cycle duration and protein aggregate number were measured and found similar in the presence and absence of continuous illumination.

To quantify the total number of aggregates and to visualize small fast-moving aggregates and fusion events, we used highly inclined and laminated optical sheet microscopy (HILO) [44] with a high laser power, on a total internal reflection fluorescence (TIRF) microscopy setup. Whereas TIRF illuminates up to 200 nm from the surface of the coverslip, HILO allowed us to image deeper in the cytoplasm, up to a depth of about 1.5 µm [44]. An Olympus-IX71 (Olympus, Tokyo, Japan) inverted microscope was used. Incidence angle of a DPSS 491 nm laser was changed to allow for excitation of the fluorophores in the sample up to 1 µm deep (1/3 of the cell volume was illuminated). Cells close to the glass surface of a MatTek dish (MatTek, Ashland, USA) were imaged, one at a time, with continuous excitation and laser power of 80% for fast imaging (200 frames/s, duration 20 s) and 10% for slow imaging (10 frames/s). An Olympus PlanApo 100×1.45 NA TIRFM objective (Olympus, Tokyo, Japan) and an Andor iXon EM+ DU-897 BV EMCCD (Andor, Belfast, UK) camera were used. Images were acquired while incubating the cells in EMM at 25°C, in order to decrease autofluorescence.

### Co-Localization Between Protein Aggregates and Subcellular Structures

Protein aggregates and subcellular structures were imaged simultaneously to test for co-localization and coordinated movement using bright field, a complementary set of fluorescent proteins (GFP, RFP, or mCherry) and dyes (Phalloidin and FM-464). We labeled protein aggregates indirectly with Hsp104-GFP or Hsp104-mCherry. Bright field was used to directly visualize cell poles and the division plane. Actin was indirectly labeled *in vivo* by expressing a calmodulin domain coupled to an N-terminal GFP (GFP-CHD) and directly labeled *ex vivo* in formaldehyde fixed cells with 2.5 µM phalloidin. Microtubules and the microtubule nucleating center (the spindle-pole body, SPB) were directly labeled using two structural components, atb2-mCherry and sid4-RFP, respectively. The nuclear membrane was directly labeled with bqt4-mCherry, an integral nuclear membrane protein. Incubating cells in 1 mM FM-464 for 10 h resulted in the direct labeling of several lipid structures [45] (cellular membrane, vacuoles, endosomes, and the Golgi complex).

## Supporting Information

Figure S1 Hsp104 interacts with aggregated proteins in *S. pombe*, and its disaggregase activity decreases aggregate number *in vivo*. (A) Images of cells expressing Hsp104-GFP and Hsp16-, Hsp70-, Gln1-, Gly1-, and Cts1-mCherry, respectively, under control and heat stress (40°C for 30 min). (B) Co-localization (white, marked with asterisks) of Hsp104 (green) and enzyme/chaperone puncta (magenta) was >90%. (C) Percentage of Hsp104 puncta containing a specific enzyme/chaperone or chaperone. (D) Puncta number per cell, control (white) and heat stress (grey). (E) Immunoprecipitation of Hsp104 with an anti-GFP antibody targeting Hsp104-GFP. Gln1 and Cts1 specifically co-immunoprecipitated with Hsp104 (see labels). (F) Fluorescence images of cells expressing Hsp16-GFP or Hsp70-GFP under normal conditions or upon heat stress (40°C for 1 h). Wild-type strains are compared to Hsp104 deletion strains. (G) Quantification of puncta number in cells shown in (F) (see labels). (H) Fluorescence microscopy of cells expressing mCherry-labeled aggregation-prone enzymes under control conditions, after heat stress and in a strain where Hsp104 was deleted. (I) Quantification of puncta number in cells shown in (H) (see labels). Data are shown as mean ± SEM; number of cells are given in the graphs. Thin lines are used to indicate cell boundaries; scale bars, 1 µm.
(EPS)

Figure S2 Protein aggregates move by diffusion, are not associated with the actin or microtubule cytoskeleton, and nucleate and fuse when the cytoskeleton is absent. (A) Aggregate nucleation (white stars) occurs in the cellular compartments on either side of the nucleus (old pole corresponds to the larger sister cell; new pole corresponds to the smaller sister cell). (B, Left) Average aggregate nucleation in each cellular compartment per cell cycle and average number of aggregates segregated to each sister cell and (Right) segregation of the aggregate in cells that contained only a single aggregate, to old or new pole cells. The *p* values for a *t* test are shown. (C) MSD of the aggregates shown in Figure 1B for a longer time scale (range of tens of minutes). The aggregates of different size classes seem to undergo subdiffusion for longer time scales (around 2,000s). (D) Time-lapse of Total Internal Reflected Fluorescence (TIRF) images showing Hsp104-GFP–labeled protein aggregates, in the presence and absence of cytoskeletal components, and the corresponding kymographs. On this fast time scale, small aggregates (asterisk) move, whereas large aggregates appear immobile. (E) MSD of small aggregates (corresponding to the fraction below the visibility threshold in the model, (*ν*) tracked with a time resolution of 5 ms as a function of Δt. A weighted fit to the equation *MSD = *4*D*Δ*t* + offset (green) yielded a diffusion coefficient (*D*) of 0.1 µm^2^/s. Fitting with a nonlinear equation (*MSD = v^2^*(Δ*t*)*^2^ +* 4*D*Δ*t +* offset, directed motion) yielded a worse fit (adjusted r^2^_(linear, 0–0.1 µm2)_ = 0.925 r^2^_(nonlinear, 0–0.1 µm2)_ = 0.851). (F) Small aggregates (*panel*; dashed circles) that are not visible by conventional wide-field microscopy were quantified (*graph*; the number of aggregates was multiplied by 3, as we imaged roughly 1/3 of the total cell volume with TIRF). (G, Left) Localization of Hsp104-GFP aggregates (green) with respect to subcellular structures: cell poles, bright-field image; nucleus, bqt4-mCherry; lipid vesicles, 1 mM FM-464; actin cables, calmodulin-GFP (GFP-CHD); actin patches, phalloidin 2.5 µM (formaldehyde fixed cells); microtubules, atb2-mCherry; spindle pole body, sid4-RFP. (Right) Quantification of co-localization between aggregates and the corresponding cellular structure (1 or 2 aggregates/cell, 70<*n*<320 cells). (H) Time-lapse overlay of aggregates (white, green) and subcellular structures (magenta) during the cell cycle (as described in G). The cellular structures observed do not move coordinately with the aggregates. (I) MSD (black dots) of aggregates tracked with a time resolution of 1 min grouped by size (see labels) after actin (Left) or microtubule (Right) depolymerization, as a function of Δt. A weighted fit to the equation *MSD = *4*D*Δ*t* + offset (lines) yielded a better fit than a weighted fit with a nonlinear equation (*MSD = v^2^*(Δ*t*)*^2^ +* 4*D*Δ*t +* offset, directed motion; adjusted r^2^_(linear, Lat.B, 2–5 µm2)_ = 0.950, r^2^_(nonlinear, Lat.B, 2–5 µm2)_ = 0.947; adjusted r^2^_(linear, MBC, 2–5 µm2)_ = 0.947, r^2^_(nonlinear, MBC, 2–5 µm2)_ = 0.477). (J, Left) Actin depolymerization after Latrunculin B and (Right) microtubule depolymerization after MBC treatment. The actin cytoskeleton was also disrupted upon heat stress (see labels). (K) Quantification of nucleation and fusion events in the absence of the actin or microtubule cytoskeleton (see labels). Data are shown as mean ± SEM; number of cells are given in the graphs. Thin lines encircle cells; scale bars, 1 µm.
(EPS)

Figure S3 Sensitivity test of the model parameters. (A) Parameters of the model. Data are shown as mean ± SEM; number of cells are given in the graphs. The sensitivity of two key model outputs, (B) the fraction of cells born clean at division 3 after stress, and (C) the average number of aggregates per cell immediately after stress, to variations in the parameters indicated. Sensitivity is calculated as (% change in output/% change in parameter).
(EPS)

Figure S4 Dynamics of individual protein aggregates after stress is similar to favorable conditions. (A) Aggregate movement after stress. Fusion events (cross) are shown in the kymograph. (B) MSD of aggregates after stress grouped by size as a function of Δt (for control, see Figure 3B). A weighted fit to the equation *MSD = *4*D*Δ*t* + offset (lines) yielded a better fit than a weighted fit with a nonlinear equation (*MSD = v^2^*(Δ*t*)*^2^ +* 4*D*Δ*t +* offset, directed motion, adjusted r^2^_(linear, 2–5 µm2)_ = 0.964, r^2^_(nonlinear, 2–5 µm2)_ = 0.661). Similarly to the control situation, aggregates move by diffusion after stress. (C) Quantification of co-localization of actin (GFP-CHD, green, strain MC193, *n* = 120 aggregates, 20 cells) and microtubules (mCherry-atb2, magenta, strain MC198, *n* = 132 aggregates, 20 cells) with Hsp104-associated aggregates after heat stress. (D) Quantification of nucleation and fusion events after stress in the absence of the actin (Lat.B) or microtubule (MBC) cytoskeleton. Nucleation and fusion were not affected by the absence of these cytoskeletal structures. (E, Left) Segregation of Hsp104-GFP–associated aggregates to the new (smaller sister cell) or old (larger sister cell) cell poles in the first and second division after heat stress. (Right) Quantification of aggregates segregated to the corresponding cell (see labels). (F) Correlation between aggregate number and division time (normalized by corresponding generation after stress; left, oxidative stress; right, heat stress), for cells inheriting a similar amount of aggregates at birth (n_Oxidative stress_ = 67 cells, n_Heat stress_ = 108 cells, *p* values representing statistical difference between cells carrying one aggregate (1) or more than one aggregate (>1): *p<0.05, **p>0.05). There was no significant difference in the division time of cells born with different aggregate number. (G) Size distributions of aggregates per cell immediately after stress (t_0_), in the second division after stress (Div._2_), and in the control population (model and experiments, see legend). (H) Number of fusion events during the first cell cycle after stress is plotted against the number of aggregates present in the cell immediately following stress from the experiment (*n* >30 cell cycles for each point, green) and model (black). The increase in aggregate number correlates with an increase in fusion events per cell cycle. (I) Aggregate segregation asymmetry at the first two divisions after heat stress (T = 40°C, 30 min), |*n*_1_−*n*_2_|, as a function of the number of aggregates at division (*n*_1_+*n*_2_), where *n*_1_ and *n*_2_ are the numbers of aggregates in the sister cells, in the experiment and the model. (J) Percentage of cells born without stress-induced aggregates, after a fixed number of divisions after stress. (K) Hsp104-GFP–labeled protein aggregates in wild-type cells and mutants under favorable growth conditions (see labels). (L) Aggregate amount per cell, in the wild-type cells and Hsp16, Hsp40, and Hsp70 deletion mutants. (M) Aggregate number distribution, per cell, in the wild-type cells and Hsp16, Hsp40, and Hsp70 deletion mutants (*p* values representing statistical difference between wild type and mutants: *p<0.05, **p<0.01). Data are shown as mean ± SEM; number of cells are given in the graphs. Thin lines encircle cells; scale bars, 1 µm.
(EPS)

Movie S1 Aggregates nucleate, fuse, and grow in the same cytoplasmic compartment during the cell cycle. Nucleation events are shown by the appearance of puncta (Hsp104-GFP, black) and fusion events occur by the merging of two puncta. Aggregates do not cross over from the cytoplasmic space on one side of the nucleus to the other during the cell cycle. The strain used for imaging was MC19 (Table S1). On the left, a bright-field image of the cells and on the right a maximum intensity projection of a z-stack of 10 images, acquired every minute. Movie is displayed at 7 fps. Time is shown in minutes; scale bar, 2 µm. (avi, 0.7 MB).
(AVI)

Movie S2 Aggregates move by diffusion in the cytoplasm. The movement of Hsp104-GFP–labeled aggregates (black dots) in short (Left, TIRF) and long (Right, conventional wide-field) time scales is shown. Fast moving small aggregates are visible, while large aggregates move slower, which is indicative of diffusive movement. The strain used for imaging was MC19 (Table S1). The movie on the left is a maximum intensity projection of five single plane TIRF images acquired at 200 fps. The movie on the right is a maximum intensity projection of a z-stack of 10 images, acquired every minute. Movies are displayed at 7 fps. Time is shown in seconds; scale bars, 2 µm (avi, 0.7 MB).
(AVI)

Movie S3 Aggregate segregation symmetry depends on the morphological symmetry of cell division. Hsp104-GFP–labeled aggregates segregate at division in cells that divide off-center (*Δpom1*). A higher number of aggregates (Hsp104-GFP, green) segregated to the larger sister. The strain used for imaging was MC75 (Table S1). An overlay between a bright-field image of the cells and a maximum intensity projection of a z-stack of 10 images acquired every 10 min is shown. Movie is displayed at 7 fps. Time is shown in minutes; scale bar, 5 µm (avi, 0.5 MB).
(AVI)

Text S1 Supporting text for experimental and theoretical procedures. (1) Supporting experimental procedures: this section contains the specific details of the experimental methods. (2) Supporting theoretical procedures: this section contains the mathematical description of the model for aggregation and aggregate segregation, including the relevant equations. A full list containing the genotype of the strains is available in Table S1.
(DOCX)

## Abbreviations

a.u.: arbitrary units
GFP: green fluorescent protein
Hsp: heat shock protein
MSD: mean squared displacement
