# Supplementary material for: Fusion of Protein Aggregates Facilitates Asymmetric Damage Segregation
Source: PLoS Biol. 2014 Jun 17;12(6):e1001886. doi: 10.1371/journal.pbio.1001886 (PMC4061010; doi:10.1371/journal.pbio.1001886)
Supplement: Text S1 — Supporting text for experimental and theoretical procedures. (1) Supporting experimental procedures: this section contains the specific details of the experimental methods. (2) Supporting theoretical procedures: this section contains the mathematical description of the model for aggregation and aggregate segregation, including the relevant equations. A full list containing the genotype of the strains is available in Table S1. (DOCX) [file pbio.1001886.s008.docx]

**Supporting information for**

**Fusion of protein aggregates facilitates asymmetric damage segregation**

Miguel Coelho, Steven J. Lade, Simon Alberti, Thilo Gross, and Iva M. Tolić-Nørrelykke

**Supporting inventory**

1. **Supporting Experimental Procedures**
2. **Supporting Theoretical Procedures**
3. **Supporting References**

## 1. SUPPORTING EXPERIMENTAL PROCEDURES

**Hsp104-GFP puncta represent physiologically occurring aggregates.** To further characterize the Hsp104-GFP puncta, we tested for co-localization with other chaperones, as reported for several organisms [[1](#_ENREF_1)]. The small heat shock protein Hsp16 and an Hsp70 chaperone were present in puncta in cells with indigenous or GFP-tagged Hsp104 (Figures S1A and S1B). We observed also that Hsp104-GFP was present in puncta of glutamine synthetase 1 (Gln1), cytidine triphosphate synthase 1 (Cts1), and also threonine aldolase (Gly1), which represent metabolic enzymes known to form aggregates [[2](#_ENREF_2),[3](#_ENREF_3)] (Figures S1A-D). Moreover, Gln1 and Cts1, independently co-immunoprecipitated with Hsp104 (Figure S1E), suggesting a physical interaction between the disaggregase and the aggregated metabolic enzyme. While the fraction of Hsp104-associated aggregates containing Gln1 and Cts1 did not change after stress, we observed an enrichment of Gly1 in Hsp104 puncta (Figure S1C). This change in aggregate composition most likely reflects a higher aggregation propensity of heat-denatured Gly1 in comparison with Gln1 and Cts1 (Figure S1A). The number and size of Hsp104 aggregates *in vivo* depends on Hsp104 activity, since the disruption of Hsp104 by a knock-out approach resulted in a higher number of pncta and a larger size, both for favorable growth conditions and stress conditions (Figures S1F and S1G). This result is consistent with results from *S. cerevisiae* [[4](#_ENREF_4)]. The number of puncta formed by aggregation-prone enzymes was similar in cells with indigenous and GFP-tagged Hsp104 (Figures S1H and S1I). We conclude that Hsp104-GFP is an active disaggregase and a reliable marker to analyze the *in vivo* dynamics of protein aggregates in *S. pombe*. Taken together, our data show that Hsp104 puncta are composed of aggregated proteins and chaperones, as reported for other organisms [[1](#_ENREF_1)].

**Cell death is correlated with accumulation of Hsp104 aggregates.** Under favorable growth conditions, a fraction of cells died (0.70±0.14%, *n*=7568). Cell death was phenotypically identified by the shrinkage of the cell to an irregular shape and darkening of the cytoplasm. Death typically occurred immediately after cell division. After these morphological changes occurred, cell growth stopped, together with the movement of lipid droplets in the cytoplasm. As previously described, these changes in morphology result in the internalization of acridine orange, a dye that marks cellular viability, indicating metabolic death [[5](#_ENREF_5)]. Cells that were identified as dead did not exhibit recovery of cell shape nor growth during the remaining divisions of the colony, while surviving cells grew and divided uninterruptedly until the end of the experiment (10h to 20h). Cells in the vicinity of the dead cell did not exhibit death or growth defects, indicating that death was not a localized response to changes in the growth media.

**Co-immunoprecipitation of Hsp104 and aggregated proteins.** Hsp104 co-immunoprecipitation experiments were carried out with non-filtered cell lysates, using beads labeled with an antibody directed against GFP (GFP-trap, Chromotek, Germany). As a control for specificity, beads were incubated with strains in which Hsp104 was not labeled with GFP. We confirmed that the anti-GFP beads did not cross-react with the mCherry epitope used for the targets (mCherry-target was pulled down in strains where Hsp104 was not labeled with GFP). To exclude that Hsp104 (bait) or the aggregation-prone enzymes (targets) bind to the beads non-specifically, we incubated samples with “blank” beads without antibody.

**Aggregate nucleation and fusion**. Aggregate nucleation was defined by the appearance of a single punctum with a detectable size (wide-field fluorescence imaging). The puncta have a minimum area of 0.066µm^2^ (2x2 pixels), appearance of puncta below this size was not considered as nucleation events. Furthermore, after nucleation, the puncta persisted in time and increased in area and intensity. Aggregate fusion events were defined as two aggregates moving into close proximity (contact event, when the distance between the centers of aggregates was smaller than the sum of their radii), moving together and later merging into a larger aggregate. Failure of fusion was observed when upon contact, the aggregates move away from each other and do not merge, retaining their original size.

**Aggregate movement and estimation of diffusion coefficients.** The x and y coordinates of aggregates in maximum intensity projections were obtained with a custom-made software for particle tracking (Alexander Krull, MPI-CBG, Dresden, Germany). To analyze the aggregate movement, a weighted fit of the equation *MSD* = 4*D*∆*t* + offset was used. Fitting with a non linear-equation (*MSD=v^2^*(∆*t*)*^2^ +*4*D*∆*t +*offset) with a directed movement component (*v^2^*(∆*t*)*^2^* ) yielded a worse fit for the different size classes, compared to the linear equation representing diffusion. The goodness of fit was estimated with the r^2^ adjusted for the degrees of freedom (number of parameters in the fitted equation). In the figure captions, we give r^2^ for a representative example, the largest-sized class of aggregates (2-5µm^2^).

The diffusion coefficient decreased with increasing aggregate area, as expected for Stokes diffusion (Figure 1B, inset). Aggregates did not co-localize with cytoskeletal or lipid structures during the whole cell cycle (Figure S2G and Figure S2H) and their movement was diffusive also after microtubule or actin depolymerization (Figures 1A, S2I). Importantly, aggregate fusion was not prevented by actin or microtubule depolymerization (for cells treated with the actin depolymerizing drug Latrunculin B or the microtubule depolymerizing drug MBC, 47/58 or 48/57 contacts resulted in fusion, respectively; Figure S2K). Thus, we found no evidence for active transport or anchoring of aggregates to cytoskeleton or lipid structures.

**Quantification of aggregate number and amount.** The number of aggregates in each cell was counted in a maximum intensity projection using Fiji, a freely distributed software. We assumed that puncta overlapping in z-stacks (same xy coordinates in consecutive z-slices) that moved coordinately correspond to a single aggregate. For Hsp104-GFP intensity quantification, the images were projected using the average intensity projection method (Fiji), which sums the intensity of pixels along the z-axis, at each time point. The area and total intensity of segmented puncta (Hsp104-GFP aggregates) was automatically measured using the “magic wand” function, using a fixed threshold.

**2. SUPPORTING THEORETICAL PROCEDURES**

## Modeling of the aggregate behavior

Gillespie algorithm*.* Model simulations were performed using a variant of the Gillespie algorithm[[6](#_ENREF_6)]. In general, Gillespie algorithms simulate a set of possible reactions that have (state-dependent) rates *R_i_*. At each time step the algorithm generates two random numbers, *q*_1_ and *q*_2_ between 0 and 1. The time counter in the simulation is then advanced by a time step ln(1/*q*_1_)/*α*, where $\alpha=\sum_{i} R_{i}$ . After this time a reaction *µ* is chosen such that $\sum_{i=1}^{\mu-1} R_{i}<q_{2}\alpha\leq\sum_{i=1}^{\mu} R_{i}$. The state of the system is updated according to *µ* and the above steps repeated.

Efficient Gillespie algorithm for aggregation. To model the fusion (fus) and generation (gen) of aggregates during the cell cycle, we use a version of the Gillespie algorithm modified to increase the efficiency of aggregation simulations (Figure 2A). We follow exactly the algorithm of Laurenzi et al. [[7](#_ENREF_7)] with the exception that we also model a generation rate *r*. The algorithm simulates the changes over time of a size distribution denoted by an index *i*, *i* = 1, …, *N* where *N* is a sufficiently large, pre-allocated array length. In the simulation, there are *n_i_* aggregates of mass (size) *m_i_*. We demand that always *m*_1_ = 1, but otherwise the *m_i_* can refer to any size aggregate as required by the algorithm. The algorithm also maintains and updates a matrix *K_ij_* = *K*(*m_i_*, *m_j_*), where *K*(*m_i_*, *m_j_*) is the reaction kernel given by Equation 1 of the main text, and a matrix of reaction rates *R_ij_* where *R_ij_* = *n_i_(n_i_ – 1)K*(*m_i_*, *m_i_*)/2 if *i* = *j* and *R_ij_* = *n_i_n_j_K*(*m_i_*, *m_j_*) otherwise. Since *R_ij_* and *K_ij_* are both symmetric, only the lower (or upper) triangular part of the matrix (including the diagonal) needs to be recorded. The algorithm then proceeds as follows:

1. The size distribution specified by the arrays *n* and *m* are given initial values (as detailed in the next section). The matrices *K* and *R* are calculated accordingly.
2. The partial rate sums $p_{i}=\sum_{j=1}^{i} R_{ij}$ and the total rate sum $\alpha=r+\sum_{i} p_{i}$ are calculated, where *r* is the parameter giving the generation rate of unit-size aggregates. Note the limits of the sum to give *p_i_* ensures only the lower triangular part of *R_ij_* is sampled.
3. Independent random numbers *q*_1_ and *q*_2_ are generated from a uniform distribution on the interval (0,1].
4. The time counter *t* is incremented by ln(1/*q*_1_)/*α*.
5. If *q*_2_*α* > *α* – *r* then generation of a unit-size aggregate occurs. *n*_1_ is incremented by 1 and the algorithm then jumps to step 9.
6. Otherwise, the first participant *x* in the fusion event is chosen such that $\sum_{i=1}^{x-1} p_{i}<q_{2}\alpha\leq\sum_{i=1}^{x} p_{i}$. This can be performed by setting a counter to zero and recording the number of elements of *p_i_* required to increment it past *q*_2_α.
7. The second participant *y* in the fusion event is chosen such that $\sum_{i=1}^{x-1} p_{i}+\sum_{j=1}^{y-1} R_{xj}<q_{2}\alpha\leq\sum_{i=1}^{x-1} p_{i}+\sum_{j=1}^{y} R_{xj}$. Similarly, this can be performed by setting a counter to $\sum_{i=1}^{x-1} p_{i}$ and recording the number of elements of *R_xj_* (while increasing *j*) required to increment it past *q*_2_α.
8. The aggregate numbers for the species participating in the fusion event are updated as follows. *n_x_* and *n_y_* are decremented by 1. If there already exists a daughter index *d* such that *m_d_* = *m_x_* + *m_y_* then *n_d_* is incremented by 1. If no such *d* exists then a *d* is chosen such that *n_d_* = 0 (that size class is empty); *m_d_* is then set to *m*_x_ + *m_y_* and *n_d_* to 1. In this case we also recalculate row *d* and column *d* of the kernel matrix *K_ij_* to reflect the new mass assigned to *m_d_*.
9. We recalculate the rows and columns of *R_ij_* corresponding to any *n*_i_ that have changed. For a generation event, this involves re-calculating column 1. For a fusion event, this involves re-calculating rows and columns *x*, *y* and *d*.
10. Return to step 2 and repeat until the time counter exceeds the length of the simulation. Since all rate parameters are expressed as rates per cell cycle, the algorithm is terminated when *t* = 1.

An advantage of this algorithm is that the kernels and reaction rates do not need to be re-calculated for all possible interactions at every time step. Another advantage is that the arrays and matrices only need to be as large as the number of different types of aggregates in the compartment, not as long as the size of the largest aggregate of the compartment, which under high fusion rates could be comparatively large. This algorithm does not model the decrease in fusion rates that may occur as a cell grows in volume during a cell cycle.

The complete aggregation algorithm*.* The above algorithm simulates the changes over time of a size distribution within one compartment. This is embedded within another algorithm that controls the division of cells and segregation at division as follows:

1. Initialize a single compartment to a size distribution consisting of a small number of aggregates of mass (size) 1. We assume that aggregates of size<2 are not detectable experimentally due to their small size and fast diffusion, or to the inhability of our fluorescent marker (Hsp104-GFP) to bind to a single aggregated protein (gray aggregates in Figure 2A). Use the above Gillespie algorithm to evolve this size distribution over one cell cycle.
2. Split the existing compartments (in the first generation, the single compartment) each into two. This is performed by assigning the mother *m_i_* array to both daughter compartments, and for every *i* assigning to one of the daughters *B_i_*(*n_i_*, 1/2) aggregates and the other daughter the remaining *n_i_* – *B_i_*(*n_i_*, 1/2) aggregates. Here *B_i_*(*n_i_*, 1/2) denotes a random variate drawn from a binomial distribution with success probability 1/2 and number of trials given by the mother’s number of aggregates *n_i_*.
3. Use the above Gillespie algorithm to evolve the size distribution in each compartment over one cycle.
4. Repeat steps 2 and 3 for as many generations as desired. The number of compartments will grow exponentially.
5. Post-process the results to allocate compartments into cells, as sketched in the scheme below.

The specific quantities being recorded during a simulation run depended on the simulations being performed. In steady-state simulations, 15 generations of compartments were typically simulated. This allows for the compartments to reach a steady state and to obtain a large number of compartments over which to calculate statistics. In these steady-state simulations, the numbers of fusion and nucleation events over the last cell cycle were recorded, along with the size distributions at the end of the last cell cycle. Due to compartmentalization, the size distribution in a compartment at division corresponds to the size distribution of an entire cell at birth in the next generation. For some results, to get good statistics the 15-generation simulation was repeated multiple times and an ensemble average was performed. In all graphs, the standard errors for the model are smaller than the size of the data marker. When simulating the response to stress, the generation rate *r* was set to a large value for one cell cycle, we skipped cell division, and then *r* was set to the unstressed value and another cell cycle was simulated, then cell divisions were resumed for as many cell cycles as required.


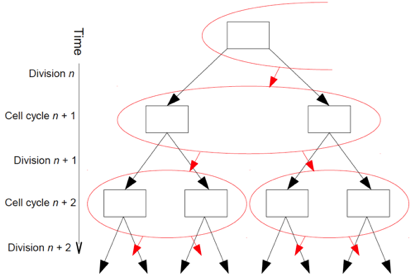


Simulation of compartmentalization. In the simulation, each cell (red oval) contains two compartments (black boxes). Aggregation and nucleation take place in each compartment in isolation. At cell division, each aggregate is randomly assigned to one of two new compartments in a daughter cell.

## Comparing the stochastic aggregation model with experimental results

The model predicts small aggregates to be common and large aggregates to be rare, which was also observed experimentally (Figure 2B). For a wide range of parameters, the model shows that the number of fusion and nucleation events per cell cycle increases and decreases with the number of aggregates at cell birth, respectively (Figures S3B and S3C). We observed the same trend experimentally (Figures S3B and S3C). Using these results (Figures 2B-D), we fitted the parameters of the model (Figure S3A). These results support the suitability of the model and its parameters for describing protein aggregate behavior.

The value of the fusion rate *k* used obtained by fitting the control data in the model can be validated by several alternative means. First, we can validate the form of the Brownian kernel (Equation 1 in the main text) that was used. This kernel assumes Stokes diffusion, namely normal diffusion of each size class of aggregates and where these diffusion coefficients follow the Stokes law. These conditions were observed experimentally (Figure 1B and its inset, respectively). Second, we can derive a quantitative estimate of *k* based on the observed normal diffusion as follows. Comparison against a mathematical derivation of the Brownian kernel [21] leads to , where *T* is the duration of a cell cycle, *V* is the typical volume of a compartment, and *a* is the fit to the measured diffusion coefficients (Figure 1B, inset) such that *D* = *a*(area)^-1/2^. Fitting *D* = *b*(area)^-1/2^ to the measured diffusion coefficients gave *b* = (1.2 ± 0.4) × 10^-4^ µm^3^/s, where the estimated uncertainty includes contributions from both the uncertainty from the fit and the uncertainties on each data point (which in turn come from the mean squared displacement fit). Assuming that aggregates are approximately spherical, with the average cross-sectional area experimentally observed for that area bin, gives *k* = (3.3 ± 1.1) µm^3^/s. Using a cylindrical compartment 7 µm long and 1.5 µm in radius and a cell cycle of 140 minutes, we obtain *k* = (0.62 ± 0.36) events/cell cycle. This value is similar to the *k* = 0.35 events/cell cycle used in the model. Third, we can estimate *k* based on the results of a comprehensive investigation of diffusion in *S. pombe* [[8](#_ENREF_8)] that indicates a mean-squared displacement of approximately 10^5^ nm^2^ for a particle of 150 nm radius (measured by electron microscopy, with negligible point spread function) after 100 s. This is a relevant time scale for the fusion of aggregates in our present experiment. These measurements, using the same formula presented above, give *k* = 0.3 events/cell cycle. We conclude that the Brownian kernel and the value of k = 0.35 events/cell cycle used in the model is reasonable, given the excellent agreement with the observed aggregate dynamics and that expected by Stokes diffusion, and quantitative agreement with independent calculations. Finally, we note that although we chose the Brownian kernel here for biophysical reasons, we expect that other kernels will also be able to reproduce observations against which model output were compared. In particular, we expect the results of the model to be robust to other kernels in the Brownian kernel's scaling class *K*(*ai*,*aj*) = *K*(*i*,*j*). Amongst the many other members of this scaling class is the widely used constant kernel *K*(*i*,*j*) = *k*.

A significant number of aggregates below the visibility threshold (ν=2) in z-stack measurements were found both in the model and in TIRFM measurements (n=4 in the model versus n=25±8 in TIRFM). We do not expect a quantitative agreement between these numbers because it is possible that in TIRFM measurements, in addition to Hsp104-GFP hexamers bound to aggregated proteins, we also observe individual Hsp104-GFP hexamers, or even smaller oligomers, which are not bound to aggregated proteins.

## Table S1. *S. pombe* strains used in this study.

| **Name** | **Mating type** | **Genotype** | **Comments** | **Source** |
| --- | --- | --- | --- | --- |
| JB107 | h- | pom1::ura4+ ura4-D18 | Deleted for pom1, divides asymmetrically | J. Bähler |
| KS3758 | h- | nmt41:GFP-CHD::leu1+ ura4-D18 leu1-32 ade6-216 | GFP labeled calmodulin (CHD) domain for actin imaging | K. Sawin |
| L972 | h- | wild-type (sequenced) | Used in transformations | G. Roedel |
| SV03 | h+ | leu1-32 ura4-D18 | Auxotroph used in transformations | S. Vogel |
| MC19 | h- | hsp104-GFP-kanr | Hsp104 labeled with GFP | Coelho *et al*.(6) |
| MC212 | h- | gln1-mCherry-natr | Glutamine synthetase 1 with mCherry | This study |
| MC210 | h- | gln1-mCherry-natr hsp104-GFP-kanr | Glutamine synthetase 1 and Hsp104 labeled | This study |
| MC304 | h+ | cts1-mCherry-natr | Cts1 labeled with mCherry | This study |
| MC252 | h- | cts1-mCherry-natr hsp104-GFP-kanr | Cytidine triphosphate synthase 1 mCherry | This study |
| MC323 | h- | hsp16-mCherry-natr hsp104-GFP-kanr | Hsp16 and Hsp104 labeled | This study |
| MC288 | h- | ssa1-mCherry-natr hsp104-GFP-kanr | Hsp70 and Hsp104 labeled | This study |
| MC76 | h+ | hsp104::kanr ade6-M216 ura4-D18 leu1-32 | Hsp104 deleted strain | Coelho *et al*.(6) |
| MC92 | h- | hsp104-mCherry-kanr | Hsp104 labeled with mCherry | This study |
| MC257 | h- | gly1-mCherry-natr hsp104-GFP-kanr | Threonine aldolase 1 with mCherry | This study |
| MC340 | h- | gly1-mCherry-kanr | Gly1 labeled with mCherry | This study |
| MC214 | h- | gln1-mCherry-natr hsp104::kanr | Glutamine synthetase 1, Hsp104 deleted | This study |
| MC319 | h- | gly1-mCherry-natr hsp104::kanr | Threonine aldolase 1, Hsp104 deleted | This study |
| MC321 | h+ | cts1-mCherry-natr hsp104::kanr | Cytidine triphosphate synthase 1 | This study |
| MC278 | h+ | hsp16-GFP-hphr leu1-32 ura4-D18 | Hsp16 labeled with GFP | This study |
| MC107 | h- | ssa1-GFP-hphr | Hsp70 labeled with GFP | This study |
| MC267 | h+ | hsp104::ura4 hsp16-GFP-hphr leu1-32 ura4-D18 | Hsp16 labeled, Hsp104 deleted | This study |
| MC234 | h+ | hsp104::ura4 ssa1-GFP-hphr ura4-D18 | Hsp70 labeled, Hsp104 deleted | This study |
| MC75 | h- | pom1::ura4+ hsp104-GFP-kanr ura4-D18 | Hsp104-GFP in asymmetrically dividing cells | Coelho *et al*.(6) |
| MC131 | h- | hsp104-GFP-kanr bqt4-mCherry-natr | Nuclear labeling, Hsp104-GFP | This study |
| MC193 | h+ | hsp104-mCherry-kanr GFP-CHD-kanr | Actin labeling and Hsp104-mCherry | This study |
| MC198 | h- | mCherry-atb2-hph hsp104-GFP-kanr leu1 ura4-D18 | Microtubule labeling and  Hsp104-GFP | This study |
| MC335 | h- | hsp104-GFP::kanr sid4-mCherry-natr | Centrosome analog (SPB) labeling | This study |
| MC135 | h- | hsp16::ura4 hsp104-GFP-kanr ura4 | Hsp16 labeled, Hsp104 deleted | This study |

##

## 3. SUPPORTING REFERENCES

1. Olzscha H, Schermann SM, Woerner AC, Pinkert S, Hecht MH, et al. (2011) Amyloid-like aggregates sequester numerous metastable proteins with essential cellular functions. Cell 144: 67-78.

2. Narayanaswamy R, Levy M, Tsechansky M, Stovall GM, O'Connell JD, et al. (2009) Widespread reorganization of metabolic enzymes into reversible assemblies upon nutrient starvation. Proc Natl Acad Sci U S A 106: 10147-10152.

3. Noree C, Sato BK, Broyer RM, Wilhelm JE (2010) Identification of novel filament-forming proteins in Saccharomyces cerevisiae and Drosophila melanogaster. J Cell Biol 190: 541-551.

4. Parsell DA, Kowal AS, Singer MA, Lindquist S (1994) Protein disaggregation mediated by heat-shock protein Hsp104. Nature 372: 475-478.

5. Miyata M, Miyata H, Johnson BF (2000) Sibling differences in cell death of the fission yeast, Schizosaccharomyces pombe, exposed to stress conditions. Antonie van Leeuwenhoek 78: 203-207.

6. Gillespie DT (1976) A general method for numerically simulating the stochastic time evolution of coupled chemical reactions. J Comp Phys 22.

7. Laurenzi IJ, Bartels, J.D. , Diamond, S.L. (2002) A General Algorithm for Exact Simulation of Multicomponent Aggregation Processes. Journal of Computational Physics 177: 418-449.

8. Tolic-Norrelykke IM, Munteanu EL, Thon G, Oddershede L, Berg-Sorensen K (2004) Anomalous diffusion in living yeast cells. Physical review letters 93: 078102.

9. Aguilaniu H, Gustafsson L, Rigoulet M, Nystrom T (2003) Asymmetric inheritance of oxidatively damaged proteins during cytokinesis. Science 299: 1751-1753.
